# Is the variant c.422+90G→A in intron 4 of indoleamine 2, 3 -dioxygenase *(IDO)* gene related to age related cataracts?

**Published:** 2011-05-05

**Authors:** M. Mamata, G. Sridhar, K. Ravi Kumar Reddy, T. Nagaraju, T. Padma

**Affiliations:** 1 Department of Genetics, Osmania University, Hyderabad, India; 2 Department of Zoology, Osmania University, Hyderabad, India; 3 Sarojini Devi Eye Hospital and Institute of Ophthalmology, Hyderabad, India

## Abstract

**Purpose:**

To screen for sequence variations in the *IDO* gene that encodes indoleamine 2, 3- dioxygenase (IDO), the first rate limiting enzyme involved in the tryptophan catabolism which results in the production of UV filters playing a role in the maintenance of lens transparency.

**Methods:**

We conducted a case-control study to screen for sequence changes in the *IDO* gene and associated demographic risk factors in patients with nuclear (NC-110), cortical (CC-110) and Posterior sub capsular (PSC-111) cataracts in comparison to normal controls (210) from Hyderabad, India.

**Results:**

Among the cataract types studied high risk was observed for CC and PSC types in female patients, individuals with low body mass index and smoking habit. Cataract development had early onset more frequently in cases of PSC followed by CC and NC. Screening by single strand conformation polymorphism (SSCP) revealed mobility shifts in 6 of the 331 patient (3 with NC and 3 with PSC) samples which upon sequencing confirmed the presence of G→A transition (c.422+90G→A; rs4613984) in the intron downstream to exon 4 of *IDO* which was further tested by RFLP anlaysis using the HhaI restriction enzyme. Of the 6 patients, one with nuclear cataract showed homozygosity and the remaining five showed heterozygosity for the substitution. None of the control samples showed this variation.

**Conclusions:**

It is possible that the substitution c.422+90G→A; rs4613984 in an intron downstream to exon 4 of *IDO* may be related with cataract formation among the aged.

## Introduction

Cataract is an age related condition characterized by progressive opacification of the ocular lens leading to visual impairment and blindness. It accounts for an estimated 16 million cases world wide, with approximately half of all the cases originating from Africa and Asia [1]. Ultraviolet (UV) light, diabetes, aging and female gender are identified as the major risk factors for the development of cataracts [2,3]. Formation of cataract is an outcome of numerous post translational modifications of crystalline proteins in the lens involving oxidation, cross-linking, truncation, and aggregation [4]. The modified proteins accumulate in the lens with age adding color, fluorescence, and insolubility [5,6]. Protein modification may also result due to thermal or photochemical reaction of UV filters like kunurenine (KN) and 3hydroxy kynurenine (3-OHKN) [7-9] formed during tryptophan catabolism. The UV filters get covalently attached to the lens proteins, influence protein functionality and increase their susceptibility to UV light [10,11]. In normal lens, with aging, the levels of free UV filters are decreased and the proteins in the lens get modified by UV [8,12-15]. In cataract lens the levels of free and protein bound UV filters are found to be much lower as compared to normal lenses of the same age while the levels of tryptophan – the precursor of kynurenine pathway is much higher [12,14,16]. This indicates possible impairment in the catabolism of tryptophan in cataractous lenses and also degradation of UV filters under oxidizing conditions [17]. From their work on the OXYS (cataract model) and Wistar (control) rats Snytnikova et al. [17] found a dramatic change between the two strains in the content of tryptophan and kynurenine during the postnatal development. They concluded that the kynurenine pathway of tryptophan catabolism does not play a significant role in cataract development in rat lens at the stages of cataract manifestation but an imbalance in the kynurenine pathway at early stages can create a metabolic background for future cataract development. Studies on human lenses in early stages of cataract are limited and the reports available on the biochemical contents are on the developed cataracts that are surgically removed. Street et al. [16] found the levels of UV filter compounds, UV filter precursor amino acid i.e., tryptophan, tyrosine, and uric acid to differ in Indian cataract lenses when compared to control lenses. They suggested that the metabolism of large proportion of patients with cataract may be different from persons with normal lenses with possible upregulation of aminoacid transporter system in cataract patients. As the levels of kynurenine were not significantly different in cataract lenses as compared to normal lenses they also concluded that there could be a defect in the lenticular UV filter pathway at one or both the steps that convert kynurenine to 3OHKG.

The first step in the process of tryptophan catabolism leading to the production of UV filters involves the oxidative cleavage of the pyrrole ring of tryptophan to N-formyl-L-kynurenine which is catalyzed by the enzyme indoleamine 2,3 dioxygenase (IDO) [18,19]. The enzyme is widely expressed in placental trophoblast giant cells of fetal origin, epididymis, gut, lymph nodes, spleen, thymus and lungs. Overexpression of the enzyme is seen in many human diseases like different types of cancers, chronic infectious diseases, allergy and autoimmune disease etc [20-22]. Apart from its key function of downregulating, the T-cell immunity, the enzyme also acts as scavenger of free radicals generated in tissues like lens which may help to prevent lens opacification [23]. Interindividual differences in IDO expression are also reported implicating inherited variations in the gene sequence. The IDO protein is encoded by the gene designated as *IDO* or *INDO* (OMIM 147435) and is located on chromosome 8p12–11 [24]. *IDO* is a single copy gene comprising 10 exons spanning 15 kb and codes for a protein of 403 amino acids. Sequence variations are reported in the *IDO* gene in the NCBI database describing 43 variants in human *IDO* gene covering exon and intronic region and their boundaries but with no clinical associations including cataract. Arefayene et al. [25] in their study using Coriell DNA samples (48 African Americans and 48 Caucasian Americans) found 24 *IDO* variants of which 17 were in exons, introns or exon/intron boundaries while 7 were within 1.3 kb upstream of the translation start site. They identified 22 putative transcription binding sites within 1.3 kb upstream of the translation site and two of the SNPs detected were located in GATA3 and FOXC1 sites. Amani et al. [26] identified 10 SNPs; four exonic and six intronic regions of *IDO* gene in Iranian women with recurrent spontaneous abortions of which three have been registered with the NCBI single nucleotide polymorphism (SNP) database which were however not associated with recurrent spontaneous abortions.

The sequence variations in *IDO* gene may have a functional role influencing UV filter production and cataract formation. Hence, the present study was performed to screen for variations in the *IDO* gene in Indian patients with different types of age related cataracts (ARC).

## Methods

We attempted to screen all the ten exons and exon-intron boundaries of *IDO* gene for sequence variations and their possible pathogenic role leading to age related cataracts by single strand confirmation polymorphism (SSCP) followed by sequencing of samples with mobility shift. The patients studied were from among the inpatients recruited for surgery at Sarojini Devi Eye Hospital and Institute of Ophthalmology, Hyderabad, India. The type of cataract was determined by the ophthalmologists concerned following LOC- III classification [27]. A total of 331 cases ([110-Nuclear cataract [NC]; 110-Cortical cataract [CC], and 111-Posterior sub capsular cataract [PSC]) were screened along with 210 healthy normal individuals selected at random by personal contacts, by house visits and from among the employees of Government and private organizations with the provision for annual health check up. The patients and controls were explained about the purpose and outcome of the study and only those who gave their consent to participate in the investigations by providing the blood samples and demographic history were considered. The study was approved by our Institutional Ethical Committee.

### Inclusion and exclusion criteria

Only patients with primary cataracts were included in the present study and those arising due to trauma, action of toxins, inflammations and degenerative ocular diseases were excluded. In addition, patients with associated conditions like diabetes, hypertension, myopia, glaucoma, thyroid syndromes, and cataract inducing medications (like steroids) were not considered. Control subjects were also without the history of cataract, diabetes, hypertension, thyroid and other ocular diseases.

From all the patients and controls, information pertaining to sex, age, age at onset, duration of disease, type of cataract, information on habits, diet, and detailed medical history along with three generation pedigrees were collected using a proforma prepared specifically for this study.

### Procedures

Venous blood samples (5 ml) were collected from all the patients and controls in EDTA vaccutainers for isolation of DNA by rapid non enzymatic method which involves salting out of the cellular proteins by dehydration and precipitation with saturated sodium chloride solution followed by extraction with absolute alcohol [28]. Ten sets of primers were designed to screen the DNA samples for variations in exon and exon-intron boundaries of *IDO* gene (Table1). The present paper refers to the variation detected in the 5′ intronic boundary downstream to exon 4 by SSCP followed by sequencing and restriction fragment length polymorphism (RFLP) analysis. The region was amplified using 10 µl PCR mix containing 1× PCR buffer, 200 µM dNTPs (Sigma Aldrich, Schnelldorf, Germany), 0.25 units of Taq polymerase (Sigma Aldrich) and 2.5 pmols of forward (5′-CAG GAG CAA GAC TCC ATC TC 3′) and reverse (5′-GTA GTG GTA GAC ACA GCA GTC 3′) primers (Ocimum Biosolutions (India) Ltd, Hyderabad, India). The PCR conditions applied were of initial denaturation at 95 °C for 5 min, followed by 30 cycles at 95 °C for 1 min, 62 °C for 40 s, 72 °C for 1 min, and a final extension at 72 °C for 5 min. The PCR products were denatured in LIS buffer (10% sucrose, 0.01% bromophenol blue and 0.01% xylene cyanol) at 95 °C for 10 min and electrophoresed for SSCP analysis using 10% polyacrylamide gel (37.5:1) for 18 h at 100 V. Later the gels were washed with distilled water, treated with ethanol acetic acid fixative (20:1) for 10 min and stained in dark with 0.1% silver nitrate (Himedia) for 10 min. Later the stain solution was replaced by developing solution (sodium hydroxide 1.5%; formaldehyde 0.15%; sodium borohydrate 0.001%) and left for about 10 min under constant shaking for the bands to develop. The banding pattern was then recorded in the Gel documentation system (Spectronics, Westbury, NY).

**Table 1:** List of primers used to screen coding regions and exon-intron boundaries of the *IDO* gene.

**Primer sets**	**Forward (5′-3′)**	**Reverse (5′-3′)**
Exon 1	CAAAAGTGGAAGTAATTTCTCAC	GAAGTTAACTTGGCCAGGTAAG
Exon 2&3	GAAGGCAAGGCATACTATCAG	GGAAAGTTAAATGTAAATTAGATG
Exon 4	CAGGAGCAAGACTCCATCTC	GTAGTGGTAGACACAGCAGTC
Exon 5	GCTTTTTCTTTTTACCTATGTCTTACC	TGGAGTCTATTGATAAACCTACATTCA
Exon 6	GATAGTAAGGCCTGCCACAC	GTTTAGGCTCCGAAGTGATTG
Exon 7	CTGGACAACTGAGCGAGACTC	CTATTCTACACCTGGAACATTTG
Exon 8	CATTATCAGTTGTACACAACACC	GGATATTAGGGACCAACCAAG
Exon 9	GGATCATGAAATCCATCTCTTG	GTGCTTTGTAGATATCCAAATAC
Exon 10A	CAGTGAATGCTATATTGGTGATC	GCAGATGGTAGCTCCTCAGG
Exon 10B	CCTGAGGAGCTACCATCTGC	GTAATGACAGGAATGCATACAG

The PCR products showing mobility shift on SSCP were sequenced on an ABI 3100 DNA sequence analyzer (Vimta labs Ltd, Hyderabad, India) which revealed the presence of c422+90G→A substitution. This substitution created a loss of restriction site for HhaI enzyme. All the patient and control samples were digested with 2 units of the enzyme at 37 °C followed by electrophoresis in 2% agarose gel with ethidium bromide. The RFLP detected single fragment of 378 bp for AA homozygotes arising due to loss of site, two fragments of 295 and 83 bp for GG homozygotes with a single restriction site and three fragments for heterozygotes GA of 378, 295, and 83 bp in length.

## Results

The results on demographic parameters showed high risk for cataracts in females with a preponderance of 55.3% as compared to males (44.7% Table 2), the frequency being highest in cases of PSC (60.4%) followed by CC (54.5%) and NC (50.9%). The ages of patients ranged from 40 to 85 years of age and that of controls from 40 to 80 years of age. The mean ages recorded for patients was 58.7±0.23 (NC-61.3±0.23, CC-58.1±0.23, and PSC-56.6±0.22) and for controls 49.1±0.10. Considering age at onset of cataract the mean values were 57.7±0.22 for the patients in general and comparatively with delayed onset in patients of NC (60.4±0.23) followed by CC (57.5±0.23) and PSC (55.6±0.22). The frequency of cases with early age at onset (<50) was high in PSC (31.5%) followed by CC (26.4%) and NC (11.8%). The frequency of over weight patients was less in cases (7.3%) as compared to controls (30.8%). Smokers among patients were higher in frequency (55.4%) and that of alcoholics lesser (39.2%) as compared to controls (smokers 39.4%, alcoholics 47.0%). The present findings are in compliance with the earlier reports from Framingham, Beaver Dam and Barbodas Eye studies and also other studies from various geographical regions suggesting association of different types of cataracts with female sex [29-33], cigarette smoking [34-43] and low body mass index [44-46].

**Table 2:** Distribution of epidemiological parameters in different types of age-related cataracts and controls studied.

** **	**N C**		**CC**		**PSC**		**Total**		**Controls**	
**Cohorts**	**N**	**%**	**N**	**%**	**N**	**%**	**N**	**%**	**N**	**%**
Total	110	33.1	110	33.1	111	33.8	331	-	210	-
Males	54	49.1	50	45.5	44	39.6	148	44.7	133	63.5
Females	56	50.9	60	54.5	67	60.4	183	55.3	77	36.5
Early onset	13	11.8	29	26.4	35	31.5	77	23.3	…	….
Late on set	97	88.2	81	73.6	76	68.5	254	76.7	….	…..
Familial	29	26.4	22	20.0	25	22.5	76	23.0	39	18.8
Non-familial	81	73.6	88	80.0	86	77.5	255	77.0	171	81.3
Smokers	29	53.7	28	56.0	25	56.9	82	55.4	53	39.4
Non-Smokers	25	46.3	22	44.0	19	43.2	66	44.6	80	60.6
Alcoholics	24	44.4	17	34.0	17	38.6	58	39.2	62	47.0
Non-Alcoholic	30	55.5	33	66.0	27	61.4	90	60.8	71	53.0
Over wt	6	5.5	8	7.3	10	9.0	24	7.3	88	30.8
Normal wt	104	94.5	102	92.7	101	91.0	307	92.7	45	69.2
	N/Mean/SEM.		N/Mean/SEM.		N/Mean/SEM.		N/Mean/SEM.		N/Mean/SEM.	
Age	110/61.3/0.23		110/58.1/0.23		111/56.6/0.22		331/58.7/0.22		210/49.1/0.16	
Age at onset	110/60.4/0.23		110/57.1/0.23		111/55.6/0.22		331/57.7/0.22		—/—/—	
BMI	110/21.8/0.23		110/21.6/0.23		111/21.9/0.22		331/21.7/0.23		210/23.4/0.16	

Screening for sequence variations revealed 6 out of 331 patient samples with mobility shift on SSCP analysis which on sequencing detected c.422+90G→A (rs4613984) substitution in the intronic region down stream to exon 4. Of the 6 patients three were with NC (one female and two males), three with PSC (two females and a male), and none with CC. All these patients were above 55 years except for one case of PSC who was a female of 25 years. This patient reported onset of cataract 6 months prior to this study and was not having any vision defects before that. Hence this patient was considered to have early onset of age related cataract rather than of juvenile onset cataract. Only one case of NC showed homozygosity (AA) for the variation while the remaining five samples were heterozygotes (GA). None of the control samples studied showed the presence of c.422+90G→A (rs4613984) substitution neither on SSCP or RFLP analysis. We may mention here that the c.422+90G→A (rs4613984) substitution registered in the NCBI database describes 5 studies with the incidence of GG homozygotes alone among four populations viz., Europeans, two different groups of Asian population, and Sub-Saharan African population. In the 5th report (pilot_1_CEU_low_coverage_panel) allele A was found with a low frequency of 0.05. No correlation of *IDO* polymorphisms have been made with any clinical conditions in these studies. In the present study the observation of AA homozygotes and GA heterozygotes made only in patients and not in controls prompts us to consider this sequence change as a probable mutation with pathogenesis for NC and PSC types of cataracts. We have not found any studies on the *IDO* gene polymorphisms with reference to cataracts so far.

## Discussion

Indoleamine 2, 3 dioxygenase (IDO) is the rate limiting enzyme in tryptophan catabolism which oxidizes tryptophan into kynurinine, initially found to affect the defense mechanism against pathogens. The enzyme activity is shown to be associated with several diseases including autoimmune disorders, cancers, depression, altered maternal tolerance of paternal antigens and apoptosis. The enzyme catalyzes the production of UV filter molecules like kynurenine and 3OH kynurenine that affect the transparency of ocular lens because of their degradation and modification of lenticular proteins by them with aging. Large inter-individual variations observed in the activity of IDO in clinical conditions have been attributed to inherited genetic variability. Though few studies describing the frequencies of the variants in some normal populations are registered in the NCBI SNP database, and by Arefayene [25] on the African American and American Caucasians samples, only one report is published on the association of Indoleamine 2,3 dioxygenase polymorphisms with recurrent spontaneous abortions in Iranian women with negative results [26].

The present study is the first report with the c.422+90 G→A substitution in the intron downstream to exon 4 occurring in 6 cases of cataracts of which only one patient was a homozygote and remaining 5 were heterozygotes for the change detected. Since none of the control samples were detected to harbor this change, we propose the variability to be associated with cataract formation. It may be mentioned here that this variation reported in normal populations all showed the presence of GG genotypes and none were of AA or AG genotypes. Thus the variation c.422+90 G→A found in the present study appears to be pathogenically related to cataract development.
